# The Effects of Reduction Mammaplasty on Serum Leptin Levels and Insulin Resistance

**DOI:** 10.1155/2015/719824

**Published:** 2015-10-13

**Authors:** Hakan Uzun, Ozan Bitik, Yahya Baltu, Çiğdem Sönmez, Ayşegül Öztürk Kaymak

**Affiliations:** ^1^Department of Plastic Reconstructive and Aesthetic Surgery, Hacettepe University Faculty of Medicine, Sıhhiye, 06100 Ankara, Turkey; ^2^Department of Plastic Reconstructive and Aesthetic Surgery, Ankara Oncology Research and Training Hospital, Yenimahalle, 06200 Ankara, Turkey; ^3^Department of Biochemistry, Ankara Oncology Research and Training Hospital, Yenimahalle, 06200 Ankara, Turkey; ^4^Department of Genetics, Ankara Oncology Research and Training Hospital, Yenimahalle, 06200 Ankara, Turkey

## Abstract

*Background.* The reduction mammaplasty has been a well-executed and known procedure in which considerable amount of fatty tissue is removed from the body. The authors aimed to show the effects of the reduction mammaplasty on serum leptin levels and insulin resistance. *Methods.* 42 obese female patients who had gigantomastia were operated on. We recorded patients' demographic and preoperative data, including age, weight, height, and body mass index. Fasting serum leptin, glucose, and insulin levels were noted. Homeostasis model assessment scores were calculated. At the postoperative 8th week, patients were reevaluated in terms of above parameters assessing the presence of any difference. *Results.* Serum leptin levels were decreased postoperatively and the decrease was statistically significant. We were able to show a decrease in homeostasis model assessment score, which indicated an increase in insulin sensitivity, and this change was statistically significant. A significant correlation between body mass index and leptin change was found postoperatively. *Conclusion.* Reduction mammaplasty is not solely an aesthetic procedure but it decreases serum leptin levels and increases insulin sensitivity, which may help obese women to reduce their cardiovascular risk.

## 1. Background

The accumulation of excess fat is associated with an increased risk for diseases, such as type 2 diabetes mellitus, hypertension, hyperlipidemias, and cardiovascular disease [1]. Normal body weight is regulated by the presence of leptin, an adipocyte derived hormone that acts on the brain to regulate food intake. It is secreted from adipocytes, mammary glands, placenta, stomach, and skeletal muscle. Leptin, a product of the obese (ob) gene, signals the hypothalamus to reduce appetite and increase energy expenditure [2]. It is a neuroendocrine protein with biological activities such as appetite regulation, energy homeostasis, bone formation, reproductive function, and angiogenesis.

Leptin is correlated with weight; its levels increase in association with increasing weight and fat mass. In obese people, it is overexpressed as compared with nonobese patients. Recent studies have figured out that leptin may help regulate blood volume and blood pressure in healthy individuals. However, in resistant situations like obesity, elevated leptin levels may lead to hypertension, renal and cardiovascular damage [3].

Body contouring procedures, such as liposuction and abdominoplasty, have increased in demand lately due to their relative safety [4, 5]. An association between peripheral fat removing by liposuction and decreasing of leptin levels already has been demonstrated [6]. Recently, abdominoplasty has been shown to decrease serum leptin concentrations as well [7].

Insulin is a critical factor for adipocyte metabolism. It has been known that insulin stimulates leptin synthesis and hyperinsulinemic conditions induce leptin resistance and hyperleptinemia [8, 9]. It has been shown that liposuction ameliorates insulin resistance [9] but abdominoplasty has been failed to change insulin sensitivity [7].

Previous studies suggest that breast volume or more precisely breast adipose tissue is linked to visceral fat and may be an independent risk factor. The reduction mammaplasty has been a well-executed and known procedure in which considerable amount of fatty tissue is removed from the body. Its effects on serum leptin levels and insulin resistance have not been studied yet. In the present study, the authors aimed to show the effects of the reduction mammaplasty on serum leptin levels and insulin resistance.

## 2. Methods

The present study was conducted according to the Declaration of Helsinki. The study was approved by Institutional Review Board. Informed consent was obtained from each patient. We recorded patients' demographic and preoperative data, including age, weight, height, and body mass index (BMI). BMI was calculated as weight in kilograms divided by the square of height in meters. Fasting serum glucose and insulin levels were noted. Insulin sensitivity in the fasting state was assessed with homeostasis model assessment (HOMA) and calculated with the following formula: fasting plasma glucose (mmol/L) × fasting serum insulin (mU/mL) divided by 25, as described by Matthews et al. [10]. High HOMA scores denote low insulin sensitivity.

In order to measure fasting leptin levels, blood samples were divided into two aliquots and immediately stored frozen at −20°C until required for analysis. Serum leptin levels were determined by using commercially available Leptin-EASIA (DIAsource ImmunoAssays S.A., Louvain-la-Neuve, Belgium) kit that was an Enzyme Amplified Sensitivity Immunoassay performed on microtiter plate. The assay was performed according to the manufacturer's protocol. The detection limit was 0.04 ng/mL. The optical density values for the plate were determined within 30 min, using a microplate reader ELX 800 (Bio-Tek Instruments, Inc., Vermont, USA).

In total, 42 obese female patients who had gigantomastia were operated on. Patient demographics and the type of reduction mammaplasty were given in tables. At the postoperative 8th week, patients were reevaluated in terms of above parameters assessing the presence of any difference.

### 2.1. Statistical Analysis

Data were analyzed using SPSS version 22.0 (IBM statistics for Windows version 22, IBM Corporation, Armonk, New York, USA). The paired samples *t*-test was used to examine the change on the basis of repeated measurements of the dependent variables. The changes within subgroups (superomedial pedicle/inferior pedicle) were then compared with each other by the general linear model repeated ANOVA (Wilks' Lambda). Pearson's correlation test and Spearman's rho test were used to determine whether any correlation existed between variables. Quantitative variables were indicated as mean and standard deviation (±). *p* value of <0.05 reflected statistical significance.

## 3. Results

The mean age of the patients was 47,67 ± 10,90. 28 patients underwent superomedial pedicle reduction mammaplasty and 14 patient underwent inferior pedicle reduction mammaplasty (Table 1). The amount of breast tissue removed was 1.954,43 ± 505,81 g (range 870 ± 3,790 g). Preoperatively mean concentrations of serum glucose, insulin, and leptin were 5,91 ± 0,97 mmol/L, 13,8 ± 11,26 mIU/L, and 25,90 ± 11,17 ng/mL, respectively. Postoperatively mean concentrations of serum glucose, insulin, and leptin were 5,20 ± 0,56 mmol/L, 11,5 ± 9,30 mIU/L, and 18,68 ± 9,30 ng/mL, respectively.

Serum leptin levels were decreased postoperatively and the decrease was statistically significant (*p* < 0.05). It means that the serum leptin levels decreased as the amount of breast reduction increased. We were able to show a decrease in HOMA score (3,11 ± 1,84 preoperatively, 2,48 ± 1,6 postoperatively), which indicated an increase in insulin sensitivity, and this change was statistically significant (*p* < 0.05). A significant correlation between BMI and leptin change was found postoperatively (Pearson's correlation coefficient *r* = 0.037, *p* < 0.05).

The clinical characteristics and the metabolic profile before and after surgery are shown in Table 2, which shows how BMI, HOMA score, glucose, insulin, and leptin levels significantly decreased after the surgery.

## 4. Discussion

Obesity is related to many diseases: in particular excessive visceral fat is an important risk factor for the development of insulin resistance and atherosclerosis and, consequently, the establishment of conditions such as type 2 diabetes, cardiovascular disease, metabolic syndrome, and also some types of cancer [11, 12]. Although the molecular mechanisms related to metabolic changes induced by excessive visceral fat have not yet been completely understood, the changes in adipokine production seem to play a crucial role in the metabolic alteration related to obesity [12].

An association between peripheral fat removing by abdominoplasty and decreasing of leptin levels already has been demonstrated [7]. Breast tissue consists of 60% adipose tissue [13] and is considered to belong to the truncal fat [14], which has been recently examined for its influence on cardiometabolic risk [15]. It has been shown that breast size in late adolescence may predict the risk of developing type 2 diabetes in middle age independent of BMI and waist circumference [16].

We hypothetically expected to show a significant relationship between amount of reduction in breasts and serum leptin levels, and we were able to show significant correlation probably because adipose tissue is the main source of leptin. Breast size has been known as an indicator of the visceral fat. It has been shown that women with greater breast size for a given BMI and waist circumference had more visceral adipose tissue than women with smaller breast volume [17]. However Schautz et al. have reported that breast adipose tissue was not associated with the proportion of visceral fat [15]. They have shown that there was also no independent association between breast adipose tissue and cardiometabolic risk factors or plasma levels of adipokines. In contrast to this study, Vinci et al. have demonstrated that reduction mammaplasty is a surgical procedure associated with a significant improvement in adiponectin level [12].

Insulin is an important mediator for adipocyte metabolism. Many studies have indicated the association between insulin resistance and serum leptin levels. These findings suggest that hyperinsulinemia induces leptin resistance and hyperleptinemia [18, 19]. In the present study, reduction mammaplasty did have a significant effect on insulin sensitivity. Our results were similar to the results of the study done by González-Ortiz et al. [20] in which they did show an improvement in insulin sensitivity after large volume liposuction in obese women although the same group had later demonstrated that there was no correlation between abdominoplasty and insulin sensitivity [7]. They concluded that insulin sensitivity is associated with visceral fat but not with subcutaneous fat. However the lifestyle change occurred after reduction mammaplasty and decrease in BMI may contribute to the increase in insulin sensitivity.

Circulating leptin levels are influenced by a variety of metabolic active factors, the most prominent of which is insulin [21]. Insulin stimulates both leptin secretion and leptin mRNA levels [22]. Therefore an increase in insulin sensitivity is expected to decrease the serum leptin levels. We think that the decrease in serum leptin levels may be related to the breast fat and the breast parenchyma removal not solely because of increase in insulin sensitivity.

We were also able to demonstrate a positive correlation between BMI and serum leptin levels as already shown by various studies. The leptin production rate in adipose tissue is directly proportional to the degree of adiposity [23]. The serum leptin concentrations in healthy men and women are positively correlated with both BMI and total body fat [24]. Although the decrease in BMI was not associated with the amount of reduction made (Pearson's correlation coefficient *r* = 0.128, *p* = 0.420), we may speculate that the lifestyle changes, which comprised an increase in exercise and a decrease in dietary intake of food, significantly contributed to the decrease in BMI.

**Figure 1:**
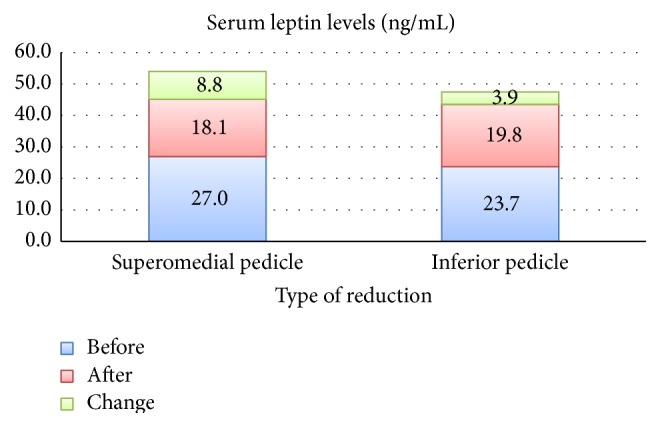
Association between serum leptin levels and type of reduction mammaplasty.

In our study, we interestingly found that the type of the pedicle chosen for the reduction mammaplasty had some relation with the serum leptin levels (Table 3). The patients for whom the superomedial pedicle was used to transport the nipple areola complex had higher decrease in serum leptin levels than the patients for whom the inferior pedicle was used (Figure 1) although the mean amount of reduction in inferior pedicle technique was higher than that of superomedial technique. The mean age of patients with superomedial technique being used was lower than that of the patients with inferior technique. Age related changes in serum leptin levels are still controversial. Isidori et al. have shown that in adult humans of different body weight serum leptin gradually declines during aging; leptin reduction is higher in women than in men, but it is independent of BMI and other age related endocrine changes [25]. However Baranowska et al. have confirmed that in elderly humans of normal body weight the plasma leptin levels were lower than in middle-aged obese persons [26]. We could not find out any study that investigates which part of the breast has more capacity to express leptin. According to the results we obtained we can speculate that inferior part of the breast has more leptin expression than the superior part. However, this finding, which may also be important for breast cancer research, should be investigated and clarified by further studies.

There were limitations that should be considered when interpreting our results. The morbid obese patients which were absent in the present study can be included in another study design. We are also aware that our small sample size could have limited our power to detect meaningful associations; however, the preliminary findings reported herein are useful for generating new hypotheses that can be investigated in larger studies.

## 5. Conclusion

Reduction mammaplasty is not solely an aesthetic procedure but it decreases serum leptin levels and increases insulin sensitivity, which may help obese women to reduce their cardiovascular risk.

## Figures and Tables

**Table 1:** The mean age of the patients and the mean amount of reduction according to type of reduction mammoplasty.

	Type of reduction mammaplasty		Total (*n*: 42)
	Superomedial pedicle (*n* = 28)	Inferior pedicle (*n* = 14)	
Age	44,79 ± 10,83	53,43 ± 8,79	47,67 ± 10,90
Amount of reduction (g)	1.844,14 ± 508,31	2.175,00 ± 437,80	1.954,43 ± 505,81

**Table 2:** The clinical characteristics and the metabolic profile before and after surgery.

	BMI (kg/m^2^)	Leptin (ng/mL)	Glucose (mmol/L)	Insulin (mIU/L)	HOMA score
Preoperative	33,94 ± 2,39	25,90 ± 11,17	5,76 ± 0,94	13,8 ± 11,26	3,11 ± 1,84
Postoperative	32,12 ± 2,08	18,68 ± 9,30	5,20 ± 0,56	11,5 ± 9,30	2,48 ± 1,6
Change (before-after)	1,82 ± 0,98	7,21 ± 4,91	0,57 ± 0,60	2,30 ± 1,23	0,63 ± 0,52
*p*	0.001	0.001	0.001	<0.001	<0.001

**Table 3:** Comparison of the serum leptin levels according to the type of reduction mammoplasty.

Serum leptin levels (ng/mL)	Type of reduction mammaplasty	
	Superomedial pedicle	Inferior pedicle
Before	26,98 ± 12,27	23,74 ± 8,54
After	18,13 ± 10,22	19,79 ± 7,32
Change	8,85 ± 4,81	3,95 ± 3,28
*p*	0.001	0.011
*p* ^1^	0.001	

*p* indicates the difference within each group.

*p* ^1^ indicates the difference between two groups.
